# Enhancing medical students comprehension through active learning: implementing the jigsaw method to improve engagement

**DOI:** 10.3389/fmed.2026.1732913

**Published:** 2026-02-09

**Authors:** Gulam Begum, B. K. M. Goud, Vijaya Marakala, Nasir A. Hamad, Smitha Elizabeth, Farida H. Khan, Anshoo Agarwal, Osama Khattak, Muhammad Amber Fareed, Khalid F. Alshammari, Vijay Bhavrao Desai, Farooq A. Chaudhary

**Affiliations:** 1 Department of Biochemistry, College of Medicine and Health Sciences, National University of Science and Technology, Sohar, Oman; 2 Department of Biochemistry, RAKCOMS, RAK Medical and Health Sciences University, Ras Al Khaimah, United Arab Emirates; 3 Department of Anatomy and Neurobiology, College of Medicine and Health Sciences, National University of Science and Technology, Sohar, Oman; 4 Department of Family and Community Medicine, College of Medicine, University of Hail, Hail, Saudi Arabia; 5 Department of Pathology, Faulty of Medicine, Northen Border University, Arar, Saudi Arabia; 6 Department of Restorative Dentistry, College of Dentistry, Jouf University, Sakaka, Saudi Arabia; 7 Department of Clinical Sciences, College of Dentistry, Ajman University, Ajman, United Arab Emirates; 8 Center of Medical and Bio-allied Health Sciences Research, Ajman University, Ajman, United Arab Emirates; 9 Department of Medicine, College of Medicine, University of Hail, Hail, Saudi Arabia; 10 School of Dentistry, Shaheed Zulfiqar Ali Bhutto Medical University (SZABMU), Islamabad, Pakistan

**Keywords:** biochemistry, cooperative learning, jigsaw method, medical education, vitamins

## Abstract

**Objective:**

The Jigsaw method is an excellent collaborative learning strategy that actively involves students, improves their problem-solving abilities, and promotes individual accountability. This study investigated the effectiveness of the Jigsaw teaching and learning technique in enhancing medical students’ knowledge of nutrition, specifically focusing on fat-soluble vitamins and B complex vitamins in medical students.

**Methods:**

A descriptive, cross-sectional, and prospective study was conducted with 176 Biochemistry students (10 males, 166 females) pursuing a Doctor of Medicine (MD) degree. Participants were conveniently sampled. The Jigsaw activity centered on “Nutrition,” with subtopics including Vitamin A, Vitamin K, B complex (B1, B2, B6), and Biotin and Niacin.

**Results:**

The results showed across all four teams, mean scores for assignment, presentation, and expert-group activities were comparable, with total scores showing minimal variation. ANOVA demonstrated significant differences in Expert Group (*F* = 11.07, *p* < 0.001) and Assignment scores (*F* = 4.24, *p* = 0.006), while Presentation scores did not differ significantly (*F* = 1.08, *p* = 0.358). Pearson correlation analysis showed strong positive associations between assignment, presentation, and total scores, indicating consistent alignment of performance components across teams.

**Conclusion:**

The Jigsaw method proved effective in boosting student engagement, comprehension, and collaborative skills in complex Biochemistry topics. The study suggests that well-planned active teaching methods like the Jigsaw model can positively impact student learning and help achieve specific learning outcomes in medical education. Our study was limited to one institution; future multi-center, longitudinal and controlled studies are recommended to validate and expand these findings.

## Introduction

Enhancing medical students’ comprehension through active learning and implementing the Jigsaw method to improve engagement is well documented in educational pedagogy (1). Moreover, cooperative learning strategies, such as the Jigsaw approach, have been recognized as among the most effective methods for promoting inclusion and fostering collaborative learning environments (2). Medical education (ME) curriculum is vast and involves multiple subjects. Students pursuing undergraduate ME encounter learning challenges owing to the complexity of the medical subjects. Moreover, medical students are expected to learn extensively about the subjects and apply them in practical and during real-life scenarios like patient treatment and management (3). Many teaching and learning (TL) methodologies have been gaining attention of ME faculties. Numerous teaching and learning (TL) approaches have drawn the attention of ME faculty members. It has been acknowledged that medical education technologies (METs), such as the traditional classroom, may not be adequate to produce competent medical graduates. Hence advanced METs like as problem-based learning, flipped classrooms, personalized learning classrooms, formative classroom evaluations methods, and others gained significance (4–7).

Jigsaw is a group based cooperative learning approach that was favored by students increasing their interest in learning (8). The jigsaw methodology was noted to be suitable for both online and offline TL activities (9). Jigsaw learning approach was first introduced in the 1970s. This TL methodology involves group learning wherein the students are further categorized into sub-groups. Further, in this approach, the students engage in learning different topics and sub-topics under the guidance of peer groups and experts (10).

Despite its student friendly approach, the jigsaw methodology was found to have mixed effects on the educational outcomes of the students that included aspects like academic achievement, motivation, self-esteem, and social wellbeing. However, it was demonstrated that the jigsaw approach had positive effects on students’ social self-esteem (11). Jigsaw method was applied among nursing students and the results demonstrated that this approach improved student’s academic achievement, skills, and attitudes (12). Conversely, Riant et al. (2) raise concerns about the conditions under which the Jigsaw method is implemented for low-achieving students. While further research is needed to consider students’ actual performance instructions, quized tests, these limitations underscore the need for teachers to ensure that the Jigsaw approach is accompanied by guided and structured instructions a factor repeatedly shown to be critical for its success.

Moreover student and teachers’ perception of jigsaw approach revealed this method was instrumental in improving the student’s communication skills and helped them overcome shyness. Teachers also reported that this method enhanced their teaching effectiveness (13).

Considering the existing literature, wherein jigsaw appeared to be a favorable TL method to accomplish the student learning outcomes (SLO’s), we implemented it in the undergraduate medical education at the College of Medical and Health sciences (COMHS), National University of Science and Technology, Sohar, Oman. The topic on Vitamins was chosen because it makes them interesting for collaborative and student-centered learning. The topic also connects metabolism, enzymology, and clinical relevance, providing a strong model for applying the Jigsaw technique. Based on this rationale, our study aimed to evaluate the effectiveness of the Jigsaw method in enhancing knowledge acquisition, improving student engagement, and exploring its integration into routine medical education as an active teaching-learning strategy.

## Materials and methods

First preclinical Doctor of Medicine (MD) students enrolled in the Biochemistry course at the College of Medicine and Health Sciences, National University of Science and Technology Sohar, Oman, participated in this descriptive, cross-sectional, and prospective study.

Ethical approval was obtained (NU/COMHS/EBC0011/2023), and participants were recruited using a convenient sampling method. A total of 176 students (10 males and 166 female students) were included representing the first preclinical year (MD2) students enrolled in MD program.

The details of the student groups, process, time, and weightage are presented in Table 1.

**TABLE 1:** Student groups and the details of the procedure.

Groups	Process	Time	Weightage
Home group	*Pre-class preparation:* Individual student assignment submission on the day of the session (Handwritten covering all the SLOs). Vitamin allotted to respective group *In-class activity:* Final group review discussion session	15 min	1.5 %
Jigsaw group	Teaching and learning from peer groups (Faculty Assessment with the Rubric)	45 min	1.5%
Expert group	*In-class group activity submission:* (Handwritten). 3 important points on each vitamin (A, K B1, B2, B6, Niacin & Biotin) learnt.	30 min	2.0%

### The jigsaw model implemented in this study

The educational philosophy underlying this strategy is a cooperative learning approach in which students actively engage in structured learning activities, enhance comprehension through peer interaction, and develop collaborative skills by working together in groups. This approach was invented by Elliot Aronson and allows each group member to focus on a single topic or specific learning outcome. The jigsaw technique follows a predetermined, sequential, linear process of student engagement, comprehension and collaboration to accomplish learning outcomes. The original Jigsaw model was used as designed, however, the implementation was contextualized to align with our curriculum structure and class size (14–16).

An orientation program on jigsaw methodology was provided to the study participants before the initiation of the study. The college follows an outcome-based education framework that incorporates active learning strategies such as Problem-Based Learning (PBL), Case-Based Learning (CBL), and Team-Based Learning (TBL) within the curriculum. Students had prior exposure to these approaches; however, the Jigsaw methodology was newly introduced to this cohort. This technique thus represented an innovative extension of the existing strategies, allowing students to build knowledge together and experience a more collaborative, peer-led form of active engagement.

The selected topic for Jigsaw activity was Vitamins—Fat soluble and B complex. The sub-topics assigned were vitamin A (VA), vitamin K (VK), B complex- B1, B2, B6 (VB), and vitamins biotin and niacin (VBiNi). The rationale for selecting this topic was that each vitamin follows a specific sequence, dietary sources, active coenzyme forms, recommended daily allowances (RDA), clinical manifestations, and deficiency disorders, which makes it easier for students to understand, master, and share with peers. This structure makes vitamins an ideal choice for self-directed, collaborative, and student-centered learning. Furthermore, the topic bridges concepts of metabolism, enzymology, and clinical relevance, making it an effective model for integrative Biochemistry learning through the Jigsaw technique.

#### Students’ division

**FIGURE 1:**
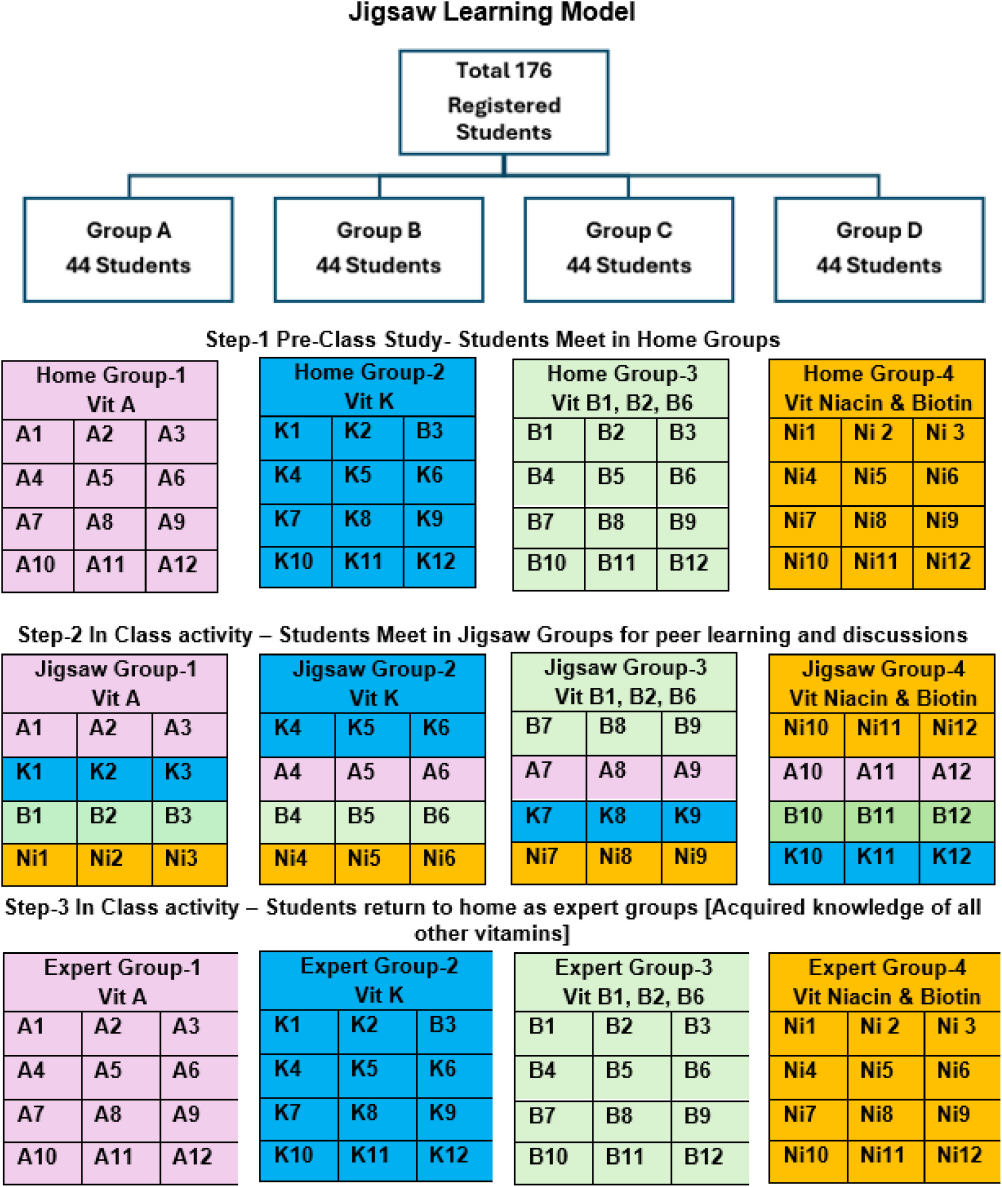
Pictorial representation of the jigsaw model.

A total of 176 students were divided into 4 groups A, B, C, and D, each comprised 40–44 students. The students were routinely divided into four practical groups (A–D) of about 41–44 students in each group. These groups rotate simultaneously across Anatomy, Physiology, and Biochemistry practical sessions. This existing organizational structure provided manageable group sizes, enabling active participation, close faculty facilitation, and effective peer interaction. Thus, the 4-day schedule reflected a practical adaptation of the regular timetable slots designated to Biochemistry practical or tutorial using our existing faculty without additional workload (Figure 1).

*Step 1:* Within each day, students were further subdivided into four home groups (VA, VK, VB, VBiNi) of approximately 10–12 members each. Individual handwritten assignments were submitted by each participant. These were narrative in format, addressing multiple levels of Bloom’s taxonomy. The questions were directly aligned with the session learning outcomes (SLOs). Student engagement was reflected in this Step (Figure 1).

*Step 2:* Three students from the different home groups VA, VK, VB, VBiNi came together to form the “jigsaw groups.” Thus, 4 jigsaw groups named 1–4 were formed. The students in the jigsaw groups included members from different home groups with different topics. The jigsaw group works together with each other discussing all the aspects of the given sub-topics, for an hour. Peer teaching among the groups enables the participants to learn other vitamins. During the process facilitators were present for each group (4 in number), who assessed the peer teaching through a rubric which was content validated and ensured internal consistency across the four groups. Cronbach’s alpha was calculated for the rubric items (α = 0.78), demonstrating satisfactory reliability for use in this context. This step reflected students’ level of comprehension.

*Step 3:* The Jigsaw group members return back to their respective home groups as an “expert group.” Each Expert group will have members who learnt all other vitamins from their peers and do a group activity of comprehending knowledge and submitting a collective assignment for all vitamin topics. Student collaborative skills were reflected during this Step.

The initial individual assignment and the final collective expert-group assignment were structured activities aligned with the session learning outcomes (SLOs) and mapped to Bloom’s taxonomy. Model answers were used for both assessments to ensure uniformity, objectivity, and consistency in scoring across all groups.

Following the procedure, the students were evaluated based on the COMHS rubric for jigsaw presentation session as shown in Table 2.

**TABLE 2:** The Jigsaw student presentation rubric evaluated cognitive understanding and affective skills.

Criteria	4 marks	3 marks	2 marks	1 mark
Demonstrates understanding of specific learning objectives relevant to the case/topic	Thorough understanding of specific learning objectives	Has good understanding of learning objectives	Has some understanding of specific learning objectives	Superficial learning of the specific learning objectives
Demonstrates the ability to present accurate information in an organized and effective manner	Excellent ability to present accurate and organized content	Presents effectively with accurate content	Not Presents effectively but content is inaccurate	Presents with inaccurate content, and/ or unorganized, ineffective manner
Demonstrates ability to clearly answer any questions raised relevant to the case/topic	Excellent ability to answer questions with accurate information	Good ability to answer questions	Unclear explanations of the doubts raised	Evasive in answering questions

## Results

The data analyzed using SPSS version 26 (*p* ≤ 0.05, the result was considered statistically significant). The data presents (Table 3) the mean and standard deviation values for four groups (A, B, C, and D) across three assessment categories: Assignment, Presentation, and Expert Group, along with the total scores.

**TABLE 3:** Scores of groups performance across different categories.

Variables	Groups	Category	N	Mean	SD
1	A	Assignment	44	10.09	1.43
2	A	Presentation	44	10.02	1.17
3	A	Expert group	44	13.5	0.88
4	A	Total	44	33.57	2.32
5	B	Assignment	44	9.98	1.58
6	B	Presentation	44	9.77	1.4
7	B	Expert group	44	13.75	0.21
8	B	Total	44	33.75	2.43
9	C	Assignment	41	10.17	0.86
10	C	Presentation	41	9.63	1.24
11	C	Expert group	41	13.85	0.15
12	C	Total	41	33.88	1.55
13	D	Assignment	42	10.9	1.27
14	D	Presentation	42	10.05	1.21
15	D	Expert group	42	13.26	0.45
16	D	Total	42	34.17	1.78

Across all four groups (A–D), mean scores for Assignment, Presentation, and Expert Group activities were comparable, with overall totals ranging from 33.57 ± 2.32 (Team A) to 34.17 ± 1.78 (Team D). The Expert Group scores showed high consistency across teams (means ≈ 13.3–13.9; SD < 1.0), indicating uniform peer-teaching performance. Slight variations in Assignment and Presentation scores reflect normal group dynamics and individual engagement differences. As shown in Table 4. One-way ANOVA and *post-hoc* interpretation showed significant differences between groups for Expert Group scores (*F* = 11.07, *p* < 0.001) and Assignment scores (*F* = 4.24, *p* = 0.006). Presentation scores did not differ significantly among the four groups (*F* = 1.08, *p* = 0.358).

**TABLE 4:** One-way ANOVA and *post-hoc* interpretation showing differences in expert group, assignment, and presentation scores among teams A–D.

Measure	Group means (A/B/C/D)	*F*-value	*P*-value	*Post-hoc* interpretation (Tukey)
Expert group	13.50 / 13.75 / 13.85 / 13.26	**11.07**	**0.000**	C > A, C > D, B > D
Assignment	10.09 / 9.98 / 10.17 / 10.90	**4.24**	**0.006**	D > B, D > C
Presentation	10.02 / 9.77 / 9.63 / 10.05	**1.08**	0.358	No significant differences

Bold values indicate statistically significant results (*p* < 0.05) based on one-way ANOVA.

Pearson correlation coefficients were used as inferential statistics to evaluate relationships between assessment components. The Pearson correlation coefficients among different performance categories for Group A are presented in Table 5. We found that there was a strong positive correlation of presentation, assignments with total scores, whereas there was a weak positive correlation of assignment with presentation in Group A group.

**TABLE 5:** Showing the correlation of various activities scores across groups.

Team	Variable pair	r (Pearson)	*P*-value	Interpretation
A	Assignment—presentation	0.333*	0.027	Weak positive
	Assignment—total	0.757**	0.000	Strong positive
	Presentation—total	0.715**	0.000	Strong positive
	Expert group—total	0.441**	0.003	Moderate positive
B	Assignment—presentation	0.336*	0.026	Weak positive
	Assignment—total	0.841**	0.000	Strong positive
	Presentation—total	0.792**	0.000	Strong positive
C	Assignment—presentation	0.130	0.418	Not significant
	Assignment—total	0.650**	0.000	Moderate positive
	Presentation—total	0.820**	0.000	Strong positive
D	Assignment—presentation	–0.013	0.935	No correlation
	Assignment—total	0.646**	0.000	Strong positive
	Presentation—total	0.711**	0.000	Strong positive
	Expert group—total	–0.026	0.872	Not significant

*Correlation is significant at the 0.05 level (2-tailed).

**Correlation is significant at the 0.01 level (2-tailed).

The Pearson correlation coefficients among different performance categories for Group B are presented in Table 5. We found a significant positive correlation of total scores with assignment and presentation variables. The Pearson correlation coefficients among different performance categories for Group C are presented in Table 5. We found again a significant positive correlation of Group total with assignment and presentation. The Pearson correlation coefficients among different performance categories for Group D are presented in Table 5. This showed again a significant positive correlation of total scores with assignment and presentation. But there was negative correlation of assignment scores with presentation, expert group but was not significant. This also seen with Group total with expert group scores and again it was not significant.

The Pearson correlation coefficients for the total scores of Groups A, B, C, and D are shown in Table 6. This showed there was moderate but significant positive correlation between scores of team C with Team A and a non-significant negative correlation between Group B total with Group A, C and D. Pearson correlation coefficients were used because all variables were continuous and met the assumptions of normality, enabling the evaluation of linear relationships between assignment, presentation, expert-group scores, and total scores.

**TABLE 6:** Correlation of total scores across groups.

Teams compared	r (Pearson)	*P*-value	Interpretation
A–B	–0.226	0.140	No correlation
A–C	0.467**	0.002	Significant positive
A–D	0.096	0.547	No correlation
B–C	–0.250	0.116	No correlation
B–D	–0.034	0.830	No correlation
C–D	0.088	0.584	No correlation

Correlation is significant at the 0.05 level (2-tailed).

**Correlation is significant at the 0.01 level (2-tailed).

## Discussion

### Educational impact of the jigsaw method

Cooperative learning is widely recognized as an effective educational strategy that enhances student engagement, comprehension, and overall academic performance. The Jigsaw method, a form of cooperative learning, actively involves students in the TL process, where learners not only take responsibility for their learning but also contribute to their peers’ understanding (14–17). The SPICES model (student-centered, problem-based learning, integrated teaching, community-based, electives, and systematic) strongly advocates for student-centered learning approaches (18). Research suggests that active engagement strategies improve long-term knowledge retention (19, 20) and enhance academic performance when incorporated into traditional passive learning methods (21, 22). The Jigsaw technique not only fosters comprehension but also promotes cooperation, effective communication, and listening skills among students (23–26). One-way ANOVA results and *post hoc* interpretation showed significant differences between groups for Expert Group scores (*F* = 11.07, *p* < 0.001) and Assignment scores (*F* = 4.24, *p* = 0.006). Presentation scores did not differ significantly among the four groups (*F* = 1.08, *p* = 0.358). These findings suggest that some teams performed significantly better than others in expert group discussions and assignments, likely due to variations in student engagement, participation levels, and group dynamics. These results align with Puppalwar et al. (27), who found that cooperative learning significantly improved student scores compared to traditional methods. Similarly, Nusrath et al. (28) suggested that the Jigsaw method enhances academic engagement and comprehension in Biochemistry education.

The correlation analysis highlights key relationships among performance categories across different Groups. In Group A, strong positive correlations were observed between total scores and both assignments (*r* = 0.757, *p* < 0.01) and presentations (*r* = 0.715, *p* < 0.01). However, a weak correlation was noted between assignment and presentation scores (*r* = 0.333, *p* < 0.05), suggesting that performance in one category may not necessarily predict performance in another. This is consistent with findings from Goolsarran et al. (29) who reported that structured cooperative learning interventions improved overall student scores but had varying impacts on individual assessment components. A similar trend was seen in Group B, where total scores had significant positive correlations with assignments (*r* = 0.841, *p* < 0.01) and presentations (*r* = 0.792, *p* < 0.01), reinforcing prior studies that emphasize the role of teamwork in academic success.

For Group C, significant positive correlations were also found between total scores and assignments (*r* = 0.650, *p* < 0.01) as well as presentations (*r* = 0.820, *p* < 0.01). This aligns with research by Bogam et al. (30), who found that cooperative learning methods helped students retain subject knowledge effectively, particularly in complex topics like Type 2 diabetes mellitus. In contrast, Group D exhibited a different pattern, with total scores significantly correlating with assignments (*r* = 0.646, *p* < 0.01) and presentations (*r* = 0.711, *p* < 0.01), but negative and non-significant, correlations between assignments and expert group scores (*r* = −0.301) and between total scores and expert group scores (*r* = −0.026). This suggests that although teamwork and engagement play a crucial role, the effectiveness of expert group discussions may depend on individual group dynamics and instructional structure, similar to observations by Dollard et al. (31) on classroom cooperation.

### Student engagement and collaborative skills

When examining total score correlations among teams, Group A and Group C exhibited a moderate yet significant positive correlation (*r* = 0.467, *p* < 0.01), suggesting some consistency in performance trends. However, Group B’s total score had a negative, though non-significant, correlation with the scores of Groups A, C, and D, implying a different performance pattern compared to the other groups. This variation supports the findings of Goolsarran et al. (29) who noted that while cooperative learning generally improves academic outcomes, individual team performance may be influenced by multiple external factors such as peer collaboration, motivation, and prior knowledge.

Studies have shown that cooperative learning strategies such as the Jigsaw method improve student comprehension, problem-solving abilities, and engagement. Research in medical and pharmaceutical education has demonstrated that the Jigsaw method enhances abstract thinking, clinical reasoning, and long-term patient care skills (32–36). Puppalwar et al. reported that students using cooperative learning performed better than those in traditional lecture-based learning and found Biochemistry more engaging through this approach (27). Similarly, Nusrath et al. recommended implementing the Jigsaw method for teaching clinical Biochemistry topics (28). Goolsarran et al. applied this method to medical postgraduate education in patient safety and found it to be both feasible and effective (29). Likewise, Bogam et al. used the Jigsaw method to teach Type 2 diabetes mellitus to first-year medical students and observed significant knowledge gains, recommending a shift from traditional lectures to cooperative learning (30).

The cooperative learning model, as observed by Dollard et al. (31) eliminates classroom competition and fosters collaboration. However, despite its numerous benefits, implementing the Jigsaw method in medical education presents logistical challenges, including time constraints and structural limitations (13, 36–39). More research is needed to explore its feasibility in diverse educational settings, particularly in Biochemistry education in regions like Oman, where studies remain limited (31–34). Our study underscores the value of cooperative learning strategies in enhancing student performance. While the Jigsaw method has proven to be effective, its implementation requires careful planning to overcome challenges associated with medical education curricula.

### Strengths

- This study demonstrates the feasible integration of the Jigsaw method into routine MD2 Biochemistry practical sessions without increasing faculty workload or requiring additional resources.
- Implementation across a large cohort (*n* = 176), supported by structured rubrics, model answers, and acceptable internal reliability (Cronbach’s α = 0.78), strengthens the robustness of the findings.
- The intervention promoted key competencies central to outcome-based medical education, including self-directed learning, peer teaching, communication, and collaboration.

### Limitations

- The study did not include a pre–post or comparator group design, as the primary focus was on feasibility and implementation within existing curricular constraints.
- Conducted at a single institution and using a foundational topic (vitamins) for first-time exposure to Jigsaw, the findings may vary in other contexts or with more complex topics.
- These aspects will be addressed in planned future multicenter and controlled studies.

## Conclusion

The results of this study indicate that cooperative learning through the Jigsaw method enhances student engagement and comprehension of Biochemistry topics. This approach fosters teamwork, communication skills, and active participation, encouraging students to take responsibility for their own learning while supporting their peers.

Well-structured teaching–learning strategies, such as the Jigsaw model, when implemented with appropriate planning and resource alignment, can positively impact student performance and knowledge retention. Integrating collaborative learning into routine teaching enables educators to achieve session learning outcomes more effectively and supports outcome-based medical education.

Future research should explore strategies to further optimize Jigsaw implementation, to facilitate its broader adoption within Biochemistry, medical, and allied health care curricula.

## Data Availability

The raw data supporting the conclusions of this article will be made available by the authors, without undue reservation.
